# Human monoclonal antibodies targeting carbonic anhydrase IX for the molecular imaging of hypoxic regions in solid tumours

**DOI:** 10.1038/sj.bjc.6605200

**Published:** 2009-07-21

**Authors:** J K J Ahlskog, C Schliemann, J Mårlind, U Qureshi, A Ammar, R B Pedley, D Neri

**Affiliations:** 1 Department of Chemistry and Applied Biosciences, ETH Zürich, Wolfgang-Pauli-Strasse 10, CH-8093, Zurich, Switzerland; 2 UCL Cancer Institute, Paul O'Gorman Building, University College London, 72 Huntley St, WC1 6BT, London, UK

**Keywords:** carbonic anhydrase, CA IX, hypoxia, phage display, tumour targeting

## Abstract

**Background:**

Hypoxia, which is commonly observed in areas of primary tumours and of metastases, influences response to treatment. However, its characterisation has so far mainly been restricted to the *ex vivo* analysis of tumour sections using monoclonal antibodies specific to carbonic anhydrase IX (CA IX) or by pimonidazole staining, after the intravenous administration of this 2-nitroimidazole compound in experimental animal models.

**Methods:**

In this study, we describe the generation of high-affinity human monoclonal antibodies (A3 and CC7) specific to human CA IX, using phage technology.

**Results:**

These antibodies were able to stain CA IX *ex vivo* and to target the cognate antigen *in vivo*. In one of the two animal models of colorectal cancer studied (LS174T), CA IX imaging closely matched pimonidazole staining, with a preferential staining of tumour areas characterised by little vascularity and low perfusion. In contrast, in a second animal model (SW1222), distinct staining patterns were observed for pimonidazole and CA IX targeting. We observed a complementary pattern of tumour regions targeted *in vivo* by the clinical-stage vascular-targeting antibody L19 and the anti-CA IX antibody A3, indicating that a homogenous pattern of *in vivo* tumour targeting could be achieved by a combination of the two antibodies.

**Conclusion:**

The new human anti-CA IX antibodies are expected to be non-immunogenic in patients with cancer and may serve as broadly applicable reagents for the non-invasive imaging of hypoxia and for pharmacodelivery applications.

Monoclonal antibodies and their derivatives are increasingly being used in anticancer therapeutic strategies for the selective delivery of bioactive agents (e.g., full immunoglobulins for Fc-mediated cell killing, drugs with cleavable linkers, radionuclides, photosensitizers, pro-coagulant factors, cytokines) to the tumour environment, thus sparing normal tissues (Payne, 2003; Adams and Weiner, 2005; Neri and Bicknell, 2005; Carter, 2006; Schrama *et al*, 2006; Schliemann and Neri, 2007; Carter and Senter, 2008). Although originally monoclonal antibodies specific to membrane antigens on cancer cells have been used for tumour targeting applications, alternative targets such as markers of angiogenesis (Schnitzer, 1998; Thorpe, 2004; Neri and Bicknell, 2005), stromal antigens (Hofheinz *et al*, 2003; Rybak *et al*, 2007; Schliemann and Neri, 2007) and intracellular proteins released at sites of necrosis (Miller *et al*, 1993; Street *et al*, 2006) are increasingly being considered. In all these cases, antibody-mediated pharmacodelivery options are particularly attractive in consideration of the fact that most conventional cytotoxic agents and many therapeutic proteins exhibit a reduced uptake at the tumour site, compared to normal organs (Bosslet *et al*, 1998; Tarli *et al*, 1999). Human or humanised monoclonal antibodies are preferred for targeting applications, as they are less immunogenic compared with rodent or chimeric antibodies (Hale *et al*, 1988; Winter *et al*, 1994).

Solid tumours are heterogenous masses and the characterisation of the different microenvironments within a neoplastic lesion provides information about the tumour structures, which can be targeted *in vivo* with intravenously (i.v.) administered monoclonal antibodies to induce a therapeutic response. In this context, the characterisation of hypoxic regions within solid tumour masses assumes a particular relevance, because hypoxic cancer cells are less sensitive to certain killing agents (e.g., radiation and cytotoxic compounds; (Weinmann *et al*, 2004; Bertout *et al*, 2008)). Hypoxic regions in tumours of experimental animal models can be assessed post-mortem by analysis of tissue sections, following i.v. administration of pimonidazole, a 2-nitroimidazole compound, which is selectively reduced and binds to intracellular macromolecules in hypoxic regions at pO_2_ < 10 mm Hg (Dearling *et al*, 2004). However, it would be desirable to complement this invasive procedure with a molecular imaging approach based on selective ligands to accessible proteins overexpressed at sites of hypoxia. Based on transcriptomic profiling of cells exposed to different oxygen concentrations, our group and others had recognised that carbonic anhydrase IX (CA IX) is one of the most overexpressed genes in hypoxic conditions (Staller *et al*, 2003; Scheurer *et al*, 2004). In parallel, *ex vivo* staining of tumour sections with monoclonal antibodies specific to CA IX had revealed staining patterns overlapping (though somewhat broader) with the neoplastic regions stained with pimonidazole (Olive *et al*, 2001; Hoskin *et al*, 2003; Sobhanifar *et al*, 2005; Jankovic *et al*, 2006; Li *et al*, 2007).

The functional contribution of CA IX to tumour growth and progression has long been debated (Pouyssegur *et al*, 2006; Brahimi-Horn *et al*, 2007). Very recently, Pouyssegur and coworkers have reported that the simultaneous invalidation of CA IX and CA XII using short hairpin RNA technology led to a substantial growth retardation in transfected LS174T colorectal cancer xenograft models, whereas the individual knock-outs yielded a substantially lower tumour growth retardation (Chiche *et al*, 2009).

Monoclonal antibodies have also been used to achieve a selective *in vivo* localisation on cells, which display a high constitutive expression of CA IX (van Dijk *et al*, 1991; Chrastina *et al*, 2003a, 2003b; Brouwers *et al*, 2004; van Schaijk *et al*, 2005), especially kidney cancer cells, in which mutations in the gene encoding the von Hippel-Lindau tumour suppressor (pVHL) lead to a constitutive HIF-1*α* activation and, as a consequence, to a strong upregulation of CA IX on all tumour cells (Wykoff *et al*, 2000; Mazure *et al*, 2004). This target was also detected as one of the most prominent accessible markers of renal cell carcinoma (RCC) in a chemical proteomic study, based on the *ex vivo* perfusion of surgically resected human kidneys with cancer using an active ester derivative of biotin, followed by capture of biotinylated proteins and mass spectrometric analysis (Castronovo *et al*, 2006).

Although hypoxic areas can be efficiently stained with CA IX antibodies *ex vivo*, at the beginning of this study, it was not known whether the same structures could be targeted *in vivo*, considering that they are typically located at a 100–200 *μ*m distance from the nearest tumour blood vessel (Kerbel and Folkman, 2002) and thus may be more difficult to reach. Indeed, although the antibody-based targeting of markers of tumour neo-vasculature is a rapid and efficient process (e.g., L19 antibody specific to the alternatively spliced EDB domain of fibronectin; (Tarli *et al*, 1999; Viti *et al*, 1999; Borsi *et al*, 2002, 2003; Berndorff *et al*, 2005; Tijink *et al*, 2006)), the tissue penetration of monoclonal antibodies to certain albuminal structures is often impaired by several factors, including molecular size (Yokota *et al*, 1992; Adams *et al*, 1998; Low *et al*, 2008), antigen barrier (Dennis *et al*, 2007; El-Emir *et al*, 2007b), and tumour interstitial pressure (Jain, 1987). In this article, we describe the generation and characterisation of two high-affinity human monoclonal antibodies (A3 and CC7) specific to the extracellular carbonic anhydrase (CA) domain of human CA IX. Both antibodies were shown to selectively recognise CA IX on the surface of tumour cells *in vitro*, in tumour sections *ex vivo* and to preferentially localise at sites of hypoxia *in vivo* following i.v. administration.

## Materials and methods

### Cell lines

Cell culture media and supplements were purchased from Invitrogen (Basel, Switzerland).

The human colorectal adenocarcinoma cell lines LS174T (CL-188, ATCC) and HT-29 (HTB-38, ATCC) were maintained in DMEM and McCoy's 5A medium, respectively, supplemented with 10% fetal bovine serum (FBS) and antibiotic–antimycotic at 37°C in an atmosphere of 5% CO_2_. The human glioblastoma cell line U87 (HTB-14, ATCC) was cultured in MEM medium, supplemented as described above. The human RCC cell line SK-RC-52 (Ebert *et al*, 1990), a kind gift of Professor E Oosterwijk, was cultured in RPMI medium, supplemented as described above. The human colorectal adenocarcinoma cell line SW1222 was maintained in MEM medium supplemented with 10% FBS, 1% glutamine and 1% non-essential amino acids.

### Cloning, expression, and purification of recombinant human CA IX

The cDNA fragment encoding the carbonic anhydrase (CA) extracellular domain (aa 120–397) of CA IX was amplified from the full-length cDNA clone IRAUp969G1273D (imaGenes, Berlin, Germany) used as template with primers BW_CA9 (5′-GGAGATCCTCAAGAACCCCA-3′) and FW_CA9_6xHis (5′-TTCCTCGAGTTAGTGATGGTGATGGTGATGACTGCTGTCCACTCCAGCAG-3′) introducing a C-terminal 6xHis-tag. A secretion sequence, required for secretion into the extracellular medium, was amplified from the construct SIP(L19)-pcDNA3.1 (Borsi *et al*, 2002) using BW_SIP (5′-GATAAGCTTGTCGACCATGGGCTGGAG-3′) and FW_SIP (5′-TGGGGTTCTTGAGGATCTCCCGAGTGCACACCTGTGGAGA-3′) primers. The resulting PCR fragments were gel-purified, assembled by PCR and cloned into vector pCEP4 (Invitrogen) by means of *Hin*dIII and *Xho*I digestion. The sequence-verified plasmid was used to transfect HEK 293 EBNA cell line using FuGENE 6 transfection reagent (Roche, Basel, Switzerland). Transiently transfected cells were cultured in DMEM supplemented with 10% FBS and selected using 200 *μ*g ml^–1^ of hygromycin (Invitrogen). The recombinant domain of CA IX was purified from supernatant by affinity chromatography on Ni-NTA agarose (Qiagen, Hombrechtikon, Switzerland) by means of the C-terminal 6xHis-tag. The purified protein was analysed by SDS–PAGE, size-exclusion chromatography using a Superdex 200 HR 10/30 column (GE Healthcare, Otelfingen, Switzerland). The specific activity of 1 *μ*g recombinant protein was measured at 400 nm by its esterase activity as a surrogate of carbondioxide hydration activity (Pocker and Stone, 1967), using 1 mM 4-nitrophenyl acetate as substrate in 100 *μ*l 50 mM Tris/SO_4_^2−^ pH 8.5. For biotinylation of recombinant CA IX EZ-Link Sulfo-NHS-LC-Biotin (Pierce, Rockford, IL, USA) was used according to the manufacturer's instructions.

### Selections of antibodies from the ETH-2-Gold library

Selections of antibodies were carried out on Immunotubes (Nunc, Roskilde, Denmark) coated with recombinant CA IX at 50 *μ*g ml^–1^ in PBS (20 mM NaH_2_PO_4_, 30 mM Na_2_HPO_4_, 100 mM NaCl, pH 7.4), as described previously by our group (Silacci *et al*, 2005).

Recombinant antibody fragments, which were positive in ELISA, in the scFv format were expressed in *E.coli* TG-1 and purified from culture supernatant by affinity chromatography using Protein A Sepharose Fast Flow resin (GE Healthcare), as described previously (Silacci *et al*, 2005).

Purified antibody fragments were analysed by SDS–PAGE and by size-exclusion chromatography on a Superdex 75 HR 10/30 column (GE Healthcare).

### Construction of the affinity maturation libraries and selections of antibodies

The first affinity maturation library of scFv(A11) was cloned by introducing sequence variability in the CDR1 loops of both heavy and light chain. Antibody residues are numbered according to (Tomlinson *et al*, 1992) and (Williams *et al*, 1996). Mutations at positions 31, 32, 33 of VH and 31, 31a, 32 of VL were introduced by PCR using the same partially degenerate primers (Eurofins MWG Operon, Ebersberg, Germany) as described previously (Villa *et al*, 2008). A single round of selection on biotinylated antigen (final concentration 10^−7^ M) was carried out eluting bound phage with 100 mM triethylamine, as described previously (Silacci *et al*, 2005). ELISA and Biacore (Otelfingen, Switzerland) screening of dissociation profiles of selected clones on a high-density coated chip yielded the antibody A3.

In the construction of the second affinity maturation library, scFv(A3) was used as template. Sequence variability was introduced in the CDR2 loops of both heavy and light chain. Mutations at positions 52, 52a, 53, and 56 of VH and at positions 50. Positions 52 and 53 of VL were introduced using partially degenerate primers (Eurofins MWG Operon), as described by (Silacci *et al*, 2006). A single round of selection on biotinylated antigen followed by a screening procedure, as described above, yielded the antibody CC7.

### Biacore analysis

Affinity measurements were performed on a Biacore 3000 instrument (Biacore). 555 RU biotinylated recombinant CA IX were immobilised onto a streptavidin SA chip (Biacore). For the real-time interaction analysis peaks representing the monomeric fractions of scFv(A3) and scFv(CC7) were collected by size-exclusion on a Superdex 75 HR 10/30 column (GE Healthcare) and injected at a flow of 20 *μ*l min^–1^ on the low-density coated antigen chip. All kinetic data were evaluated using the BIAevaluation 4.1 software (Biacore).

### Cloning, expression, and purification of antibodies in the SIP format

**Figure 1:**
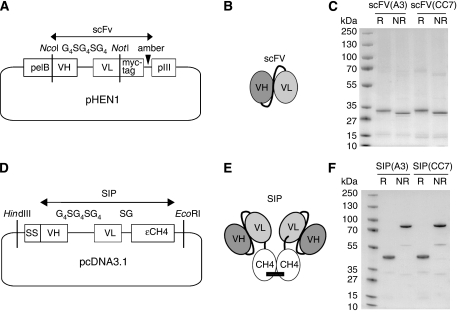
Cloning, purification and quality control of anti-CA IX antibodies. (**A**) The ETH-2-Gold phage antibody library (Silacci *et al*, 2005) was cloned into pHEN1 vector with VH and VL segments of scFvs flanked by the pelB secretion sequence, and an myc-tag followed by an amber codon and the gene-encoding pIII. (**B**, **E**) Schematic representation of the different antibody formats used in this study. ScFv fragments consist of a variable heavy (VH) and a variable light (VL) chain connected by a peptide linker, whereas the small immunoprotein (SIP) (Borsi *et al*, 2002) is a disulfide-linked homodimer of two scFv–*ε*CH4 fusions (**C**, **F**) SDS–PAGE analysis of the purified scFv(A3), scFv(CC7) and SIP(A3) and SIP(CC7), respectively, under reducing (R) and non-reducing (NR) conditions. (**D**) Cloning strategy for the SIP antibodies.

ScFvs were converted into the SIP format by cloning VH and VL into pcDNA3.1 (Invitrogen) using the same primers and strategy as described by (Silacci *et al*, 2006) (Figure 1). The plasmids were transfected into CHO-S cells (Invitrogen) using Cell Line Nucleofector Kit V (Amaxa, Köln, Germany), following the manufacturer's protocol. Transfectomas were grown in RPMI supplemented with 10% FBS and selected by addition of 500 μg ml^–1^ Geneticin (G418) (Merck Chemicals Ltd, Nottingham, UK). Monoclonal cultures were obtained by fluorescent-activated cell sorting after staining for secreted antibody, as described (Zuberbühler *et al*, 2008). After 14 days of selection, cells were brought into suspension, and cultured in Power CHO-CD 2 (Lonza, Basel, Switzerland). SIP antibodies were purified from culture medium by affinity chromatography using Protein A Sepharose Fast Flow resin (GE Healthcare), as described by (Zuberbühler *et al*, 2008).

### Fluorescence-activated cell sorting

Cells were harvested through incubation with 10 mM EDTA in PBS for 3 min at 37°C. After counting and a centrifugation step of 5 min at 1100 r.p.m., cells were re-suspended in PBS supplemented with 2% FBS to a final concentration of 5 × 10^6^ cells per ml. Cells (1 × 10^6^) were then incubated for 30 min at room temperature with 1 *μ*g ml^–1^ of SIP(A3) and SIP(CC7), respectively, in the presence of 2% FBS. For the detection, rabbit-anti-human IgE antibody (Dako, Glostrup, Denmark), followed by goat anti-rabbit IgG Alexa Fluor 488 antibody (Invitrogen) were used, diluted according to the manufacturer's recommendation. Rinsing with PBS was performed in between the incubation steps. Fluorescence-activated cell sorting (FACS) was performed on a FACSCanto equipped with FACSDiva software (BD Biosciences, Allschwil, Switzerland). A typical cell area was gated and a total of 10 000 events per sample were acquired. Omission of the primary antibody, as well as an isotype-matched control anti-lysozyme antibody, SIP(HyHEL-10) (Lavoie *et al*, 1992), at 9 *μ*g ml^–1^ were used to define background staining. Results were expressed in percentage of the maximal FACS signal. Data were analysed using FloJo software (Tree Star, Olten, Switzerland).

### Immunofluorescence on frozen tissue sections

Healthy and tumour tissue was embedded in freezing medium (Microm, Volketswil, Switzerland), snap frozen in liquid nitrogen and stored at −80°C until sectioned. Tissue sections (10 *μ*m) were first fixed for 10 min in ice-cold acetone, rehydrated with PBS, blocked with FBS and then double-stained for CA IX and CD31. SIP(A3) and SIP(CC7), used as primary binding reagents at 1 *μ*g ml^–1^, were detected with rabbit-anti-human IgE antibody (Dako), followed by goat anti-rabbit IgG Alexa Fluor 594 antibody (Invitrogen). Primary rabbit polyclonal anti-CA IX antiserum (sc-25599; Santa Cruz Biotechnology, Heidelberg, Germany) was detected with goat anti-rabbit IgG Alexa Fluor 594 antibody (Invitrogen). Primary rat anti-mouse CD31 antibody (BD Biosciences) was detected with donkey anti-rat IgG Alexa Fluor 488 antibody (Invitrogen). All commercial binding reagents were diluted according to the manufacturer's recommendation. Rinsing with PBS was performed in between all incubation steps. Finally, slides were mounted with Glycergel mounting medium (Dako) and analysed with a Zeiss Axioskop 2 mot fluorescence microscope (Carl Zeiss AG, Feldbach, Switzerland). Images were captured with an AxioCam MRC using AxioVision 4.7 image analysis software (Carl Zeiss AG).

### Multi-fluorescence microscopy

LS174T or SW1222 human colorectal adenocarcinoma cells (5 × 10^6^) were injected s.c. into the left flank of 6- to 8-week-old female MF1 nu/nu mice. Tumours were allowed to grow for 14 days to a size of typically 1–1.5 cm^3^.

All mice were injected i.v. into the tail vein with 100 *μ*g of SIP(A3) (*n*=3) or SIP(CC7) (*n*=3) 6 h before killing. To relate anti-CAIX SIP antibody distribution to tumour morphology/pathophysiology, the following parameters were studied by multi-fluorescence microscopy: (i) perfusion: the *in vivo* DNA-binding dye Hoechst 33342 (10 mg kg^−1^; Invitrogen) was injected i.v. 1 min before killing. (ii) Blood vessels: an anti-CD31 antibody was used to stain for blood vessel distribution. (iii) Hypoxia: the hypoxic cell marker pimonidazole hydrochloride (1-[(2-hydroxy-3-piperidinyl) propyl]-2-nitroimidazole hydrochloride; 60 mg kg^−1^; Natural Pharmacia International Inc., Burlington, MA, USA) was injected 30 min before killing.

Sections (12 *μ*m) were first fixed in acetone for 10 min at room temperature and blocked for 20 mins with 3% normal goat serum (Dako). Three serial sections from the same tumour were incubated with a 1:2 dilution of rat anti-mouse CD31 simultaneously with one of the following antibodies: (1) 1 : 200 dilution of rabbit anti-pimonidazole (Natural Pharmacia International Inc.), (2) 1 : 1000 dilution of rabbit anti-human IgE (Dako; to detect the injected SIP(A3) and SIP(CC7)) or (3) 1 : 10 dilution of rabbit polyclonal anti-CAIX antiserum (sc-25599; Santa Cruz). After rinsing with PBS, sections were incubated with a 1 : 200 dilution of goat anti-rat Alexa Fluor 594 antibody and goat anti-rabbit IgG Alexa Fluor 488 (Invitrogen). Sections were mounted in PBS and viewed using an AxioImager.Z1 microscope (Carl Zeiss AG), fitted with a computer-controlled motorised stage. Images were captured by an AxioCam digital black and white camera using AxioVision 4.6 image analysis software (Carl Zeiss AG). Image processing was performed as described previously (El-Emir *et al*, 2007b).

### Biodistribution in LS174T human colorectal carcinoma model

LS174T human colorectal adenocarcinoma cells (1 × 10^7^) were injected s.c. into the left flank of 6- to 8-week-old female Balb/c nu/nu mice (Charles River, Sulzfeld, Germany). Tumours were allowed to grow for 15 days to a weight of typically 200 mg. SIP(A3), SIP(CC7), SIP(HyHEL-10) and SIP(L19) were conjugated to *p*-isothiocyanatobenzyl-diethylene triamine pentaacetic acid (*p*-SCN-Bn-DTPA; Macrocyclics) in 0.1 M NaHCO_3_ buffer at pH 8.2 by incubation with a 50-fold molar excess of *p*-SCN-Bn-DTPA for 1 h at room temperature. The *p*-SCN-Bn-DTPA-SIP conjugates were purified by size-exclusion on a PD-10 column (GE Healthcare). Mass spectrometric analysis revealed ∼1.8 modifications/antibody molecule.

The *p*-SCN-Bn-DTPA-SIP conjugates were labelled with ^177^Lu (Perkin Elmer, Schwerzenbach, Switzerland) in a 125 mM ammonium acetate buffer pH 5.5 for 1 h at room temperature, as described by (Brouwers *et al*, 2004). Subsequently, free ^177^Lu was complexed by addition of 2.5 mM EDTA. The radiolabelled antibody preparation was purified by size-exclusion on a PD-10 column (GE Healthcare) and eluted with PBS. For all preparations, the rate of radiolabel incorporation and the immunoreactivity were determined, as described (Tarli *et al*, 1999). The *in vivo* targeting performance of the ^177^Lu-labelled antibody preparations was evaluated by i.v. injection of 6–11 *μ*g per mouse (7–11 *μ*Ci) in 200 *μ*l into the tail vein of xenograft-bearing nude mice. Each group contained four individuals. Mice were killed 24 h after injection. Organs were excised, weighed and the radioactivity was counted. Targeting results are expressed as both tumour/organ ratios and as percentage of injected dose per gram of tissue (% ID g^−1^).

All *in vivo* studies were carried out according to Swiss regulations under a project license granted by the Veterinäramt des Kantons Zürich (198/2005)

## Results

### Isolation of A3 and CC7, two human monoclonal antibodies specific to CA IX

**Figure 2:**
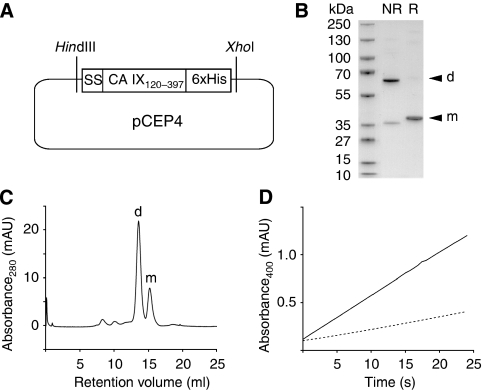
Cloning, purification and quality control of recombinant CA IX. (**A**) Residues 120–397 corresponding to the extracellular CA domain of CA IX, flanked by a secretion sequence (SS) and a C-terminal 6xHis-tag, were cloned into pCEP4. (**B**) SDS–PAGE analysis of the purified recombinant CA IX. Under non-reducing (NR) conditions the protein mainly forms a covalent homodimer (d), whereas under reducing (R) conditions the monomeric protein (m) with a molecular weight of 31.2 kDa can be observed. (**C**) Size-exclusion chromatography of the purified recombinant CA IX. The retention volume (ml) of the major peak corresponds to a covalently formed homodimer (d). (**D**) The specific activity of the recombinant protein was measured at 400 nm by its esterase activity, using 1 mM 4-Nitrophenyl acetate as substrate (solid line). Spontaneous hydrolysis of the substrate is reported as dashed line.

The CA domain of CA IX (residues 120–397) was cloned and expressed as soluble protein in HEK EBNA 293 cells (Figure 2A) and purified from the cell culture supernatant on Ni-NTA resin by means of a C-terminal 6xHis-tag. The native structure of CA IX on the cell membrane is reported to consist of cysteine-linked trimers (Pastorekova *et al*, 1992; Thiry *et al*, 2006). We observed the formation of a covalent homodimer (∼75%) and of a monomeric protein (∼25%) both by SDS–PAGE analysis (Figure 2B) and by size-exclusion chromatography (Figure 2C). The specific esterase activity of the purified protein, as measured with 1 mM 4-nitrophenylacetate and 1.6 *μ*g recombinant enzyme in 100 *μ*l of 50 mM Tris-SO_4_, pH=8.5 containing 10 *μ*M ZnSO_4_ at 400 nm, was determined to be 1 nmol min^−1^ *μ*g^−1^ (Figure 2D). This suggests that the enzyme is also catalytically active in terms of carbondioxide hydration.

The recombinant catalytic domain of CA IX was used for the isolation of human monoclonal antibodies from the ETH-2-Gold phage antibody library (Silacci *et al*, 2005). One of the clones isolated from the library (‘A11’) (Table 1), which was shown to be non-inhibitory in the enzymatic assay described above (data not shown), was affinity-matured by combinatorial mutagenesis of residues in the CDR1 loops of VH and VL domains according to a procedure recently developed by our group (Villa *et al*, 2008), yielding clone A3. Additional mutagenesis of CDR2 loops led to the isolation of the daughter antibody clone CC7 (Table 1). The A3 and CC7 antibodies were expressed as scFv fragment in *E.coli* and in small immunoprotein (SIP) format in CHO-S cells using published procedures (Borsi *et al*, 2002) and purified to homogeneity by protein A affinity chromatography (Figure 1). The scFv format is particularly suitable for affinity determination using Biacore technology, whereas the homobivalent SIP format has been shown to offer distinctive advantages for *in vivo* molecular imaging applications (Borsi *et al*, 2002; Berndorff *et al*, 2005; Olafsen *et al*, 2005; Tijink *et al*, 2006; von Lukowicz *et al*, 2007; Sauer *et al*, 2009).

### *In vitro* characterisation of A3 and CC7 antibodies

**Figure 3:**
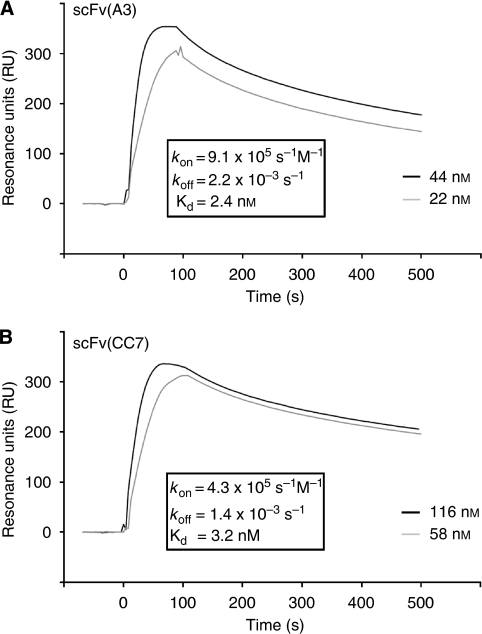
Biacore analysis of purified monomeric scFv preparations injected at different concentrations. (**A**) Binding of scFv(A3) and (**B**) scFv(CC7) to the extracellular CA domain of CA IX. Kinetic constants were calculated with the BIA evaluation 4.1 software.

The monomeric fractions of the A3 and CC7 antibodies in recombinant scFv format were isolated by size-exclusion chromatography and analysed by real-time interaction analysis on a Biacore instrument, using a microsensor chip coated with the recombinant CA domain of CA IX. Figure 3 illustrates sensograms for the two antibodies, revealing a *K*_d_ dissociation constant of 2.4 nM for scFv(A3) [*k*_on_=9.1 × 10^5^ s^−1^M^−1^; *k*_off_=2.2 × 10^−3^ s^−1^] and of 3.2 nM for scFv(CC7) [*k*_on_=4.3 × 10^5^ s^−1^M^−1^; *k*_off_=1.4 × 10^−3^ s^−1^].

**Figure 4:**
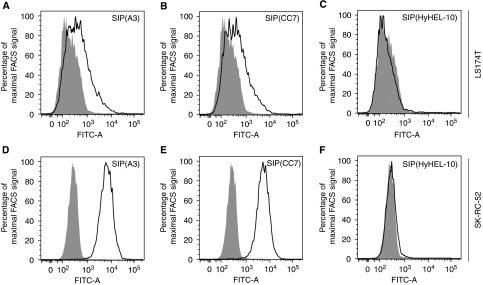
CA IX FACS histogram plots of two different human tumour cell lines. (**A**–**C**) Open curves indicate FACS histogram plots of LS174T human colorectal adenocarcinoma cells stained with SIP(A3), SIP(CC7) (both at 1 *μ*g ml^−1^) or the isotype-matched control SIP(HyHEL-10) (at 9 *μ*g ml^−1^), detected as described before. Solid curves represent FACS histogram plots of LS174T cells where the primary antibody was omitted for detection. (**D**–**E**) Open and solid curves indicate FACS histogram plots of SK-RC-52 human RCC cells stained as described for **A**–**C**.

The ability of the two antibodies in SIP format to recognise the native CA IX on the surface of tumour cells was investigated by fluorescence-activated cell sorting (FACS), in comparison to a recombinant SIP antibody of irrelevant specificity (HyHEL-10; (Lavoie *et al*, 1992). Figure 4 shows that a partial shift in the FACS profile was observed for the colorectal cancer cell line LS174T, whereas a very marked increase of cell fluorescence was detected for the pVHL-defective human RCC cell line SK-RC-52, similar to what previously reported for the clinical-stage cG250 monoclonal antibody (Grabmaier *et al*, 2000).

**Figure 5:**
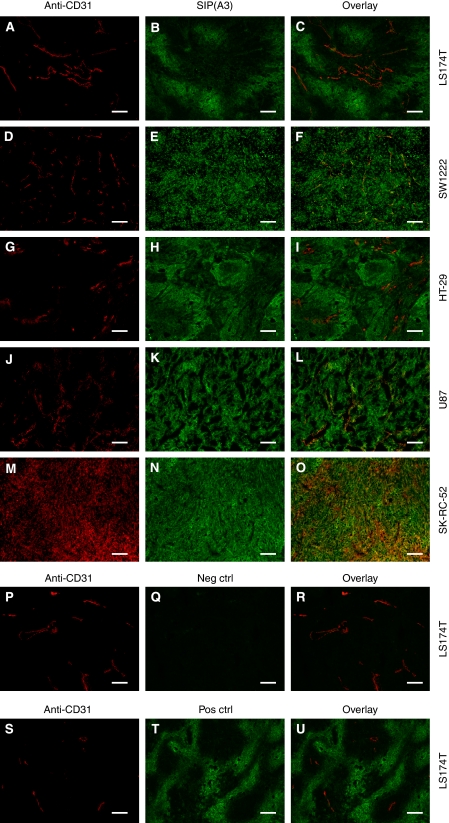
Immunofluorescence analysis performed on (**A**–**C**, **P**–**U**) human colorectal adenocarcinoma LS174T, (**D**–**F**) human colorectal adenomacarcinoma SW1222, (**G**–**I**) human colorectal adenocarcinoma HT-29, (**J**–**L**) human glioblastoma U87 and (**M**–**O**) human RCC SK-RC-52 xenografted tumour tissue sections. Red staining in the left panels represents endothelial cells (anti-CD31 staining), whereas green staining in the middle panels represents expression of CA IX ((**B**, **E**, **H**, **K**, **N**) SIP(A3) or (**T**) polyclonal anti-CA IX antiserum staining). Overlay of red and green fluorescence is shown in the panels to the right. (**Q**) Primary antibody was omitted as a negative control. Scale bar=100 *μ*m.

A3 and CC7 were then tested by immunofluorescence for their ability to detect CA IX in a panel of xenografted human tumour tissue sections. Figure 5 shows the staining obtained with A3 only, because both antibodies performed equally well. As positive control, a commercial polyclonal anti-CA IX antiserum was used. A representative positive and negative control is shown for the LS174T tumour. A strong CA IX staining (green) was detectable in the colorectal cancer models LS174T and HT-29 at a distance of ∼100 *μ*m from tumour blood vessels (red). In contrast, virtually all tumour cells in SW 1222 colorectal carcinoma, U87 glioma, and in SK-RC-52 renal cell carcinomas exhibited a strongly positive CA IX staining. However, although SW 1222 and U87 exhibited a defined vascular structure, CD31 staining in SK-RC-52 tumours yielded an irregular staining network. Other cancer types displayed a weaker level of CA IX staining (e.g., NCI-H460 human non-small cell lung carcinoma), whereas MCF-7 breast tumours and Ramos lymphomas were essentially negative (data not shown).

### A3 and CC7 selectively target hypoxic tumour regions *in vivo*

To investigate whether the new human anti-CA IX antibodies were able to selectively localise to the antigen in tumours, following i.v. administration in the tail vein, we used both fluorescence microscopy and radioactivity-based detection methods. As mouse models of human cancer, we chose LS174T and SW1222 tumours: two colorectal cancer models, which have previously been extensively studied using monoclonal antibodies specific to the carcinoembryonic antigen (El Emir *et al*, 2007a; Fidarova *et al*, 2008) and with the vascular-targeting anti-EDB antibody L19 (El-Emir *et al*, 2007b).

**Figure 6:**
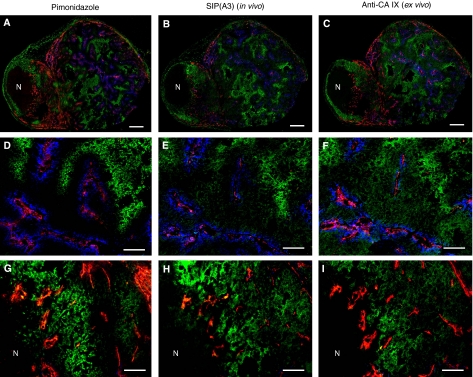
Multi-fluorescence microscopy analysis in LS174T xenograft-bearing mice. (**A**,**B**) Representative overlays of multiple digital fluorescence images of a LS174T tumour injected with pimonidazole (30 min before killing) and SIP(A3) (6 h before killing) demonstrating perfusion with Hoechst 33342 (1 min before killing) (blue), blood vessel staining (red), pimonidazole binding (green, left panel) and CA IX targeting by SIP(A3) (green, middle panel). (**C**) *ex vivo* staining of an adjacent tumour section with a polyclonal anti-CA IX antiserum. (**D**–**F**) Higher magnification images of mainly perfused areas of the corresponding tumour sections. (**G**–**I**) Higher magnification images of mainly CA IX-positive areas of the corresponding tumour sections. (**A**–**C**) Scale bar=500 *μ*m and (**D**–**I**) scale bar=100 *μ*m. N indicates necrosis.

Figure 6 shows representative results of a multi-colour fluorescence microscopy analysis of serial sections from LS174T tumours, following i.v. administration of SIP(A3) (6 h before killing of the mice), of pimonidazole (30 min before killing) and Hoechst 33342 (1 min before killing). The low magnification images (top panels) yield an informative impression of the heterogeneity of these tumours. One can recognise areas with dense vascular structures (red), some of which are well perfused (blue). The structures targeted *in vivo* by SIP(A3) closely match those stained *ex vivo* for pimonidazole modification, confirming that these hypoxic areas could be reached by the i.v. administered antibody. Importantly, these areas are superimposable to the structures stained *ex vivo* with a polyclonal anti-CA IX antiserum, thus indicating that CA IX-positive areas of the tumour could be reached by our reagent. A higher magnification view (bottom panels) shows details of well perfused areas with no detectable CA IX expression, as well as tumour regions efficiently targeted by the A3 antibody. Similar results were obtained with SIP(CC7) (Supplementary Figure).

**Figure 7:**
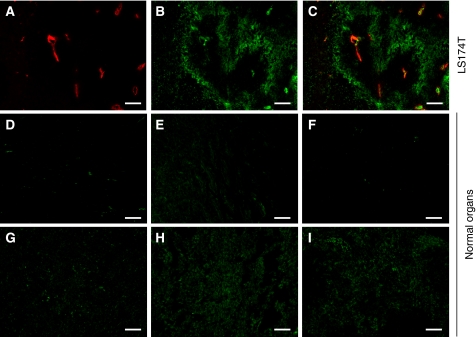
*In vivo* immunofluorescence analysis in LS174T xenograft-bearing mice. (**A**) Endothelial cells of tumour blood vessels are shown in red (*ex vivo* anti-CD31 staining). (**B**) Green fluorescence staining represents CA IX targeting in the tumour 6 h after i.v. administration of SIP(A3) detected with immunofluorescence techniques. (**C**) Overlay of red (blood vessels) and green (targeted CA IX) staining in the tumour. (**D**) Heart, (**E**) intestine, (**F**) kidney, (**G**) liver, (**H**) lung and (**I**) spleen, when stained accordingly, showed a negligible uptake of SIP(A3). Scale bar=100 *μ*m.

The selective tumour targeting of the human anti-CA IX antibodies was also evaluated by immunofluoresence, comparing sections of tumours and of normal organs, 6 h after i.v. administration of SIP(A3). Figure 7 shows that a much brighter fluorescence signal was observed in tumour lesions, compared with normal organs (heart, intestine, kidney, liver, lung, and spleen).

**Figure 8:**
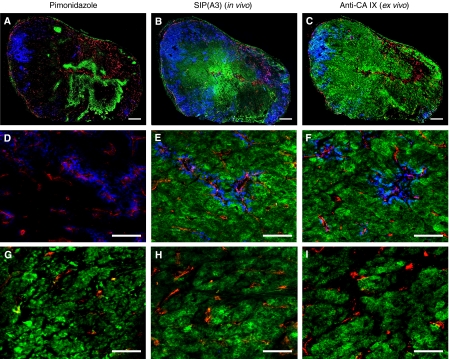
Multi-fluorescence microscopy analysis in SW1222 xenograft-bearing mice. (**A** and **B**) Representative overlays of multiple digital fluorescence images of a SW1222 tumour injected with pimonidazole (30 min before killing) and SIP(A3) (6 h before killing) demonstrating perfusion with Hoechst 33342 (1 min before killing) (blue), blood vessel staining (red), pimonidazole binding (green, left panel) and CA IX targeting by SIP(A3) (green, middle panel). (**C**) *ex vivo* staining of an adjacent tumour section with a polyclonal anti-CA IX antiserum. (**D**–**F**) Higher magnification images of mainly perfused areas of the corresponding tumour sections. (**G**–**I**) Higher magnification images of mainly CA IX-positive areas of the corresponding tumour sections. (**A**–**C**) Scale bar=500 *μ*m and (**D**–**I**) scale bar=100 *μ*m.

Figure 8 presents a similar analysis for the *in vivo* targeting of SW1222 tumours. In this model, one can observe a striking difference between external, well-perfused areas (blue) and complementary, poorly perfused hypoxic regions in the tumour core. Although an *ex vivo* analysis with the anti-CA IX antiserum yields a pattern with uniform staining intensity, the regions targeted *in vivo* by SIP(A3) mainly cluster around central vascular structures, with a gradient of staining intensity (green) which reflects a gradient in antibody diffusion. Interestingly, in this tumour model, pimonidazole staining and CA IX staining do not always overlap. Similar results were obtained with SIP(CC7) (Supplementary Figure).

To assess the efficiency of *in vivo* targeting of CA IX, we performed a comparative biodistribution analysis with SIP(A3), SIP(HyHEL-10) used as negative control and SIP(L19), a clinical-stage antibody which recognises tumour neo-vascular structures (Borsi *et al*, 2002; Berndorff *et al*, 2005; Tijink *et al*, 2006; El-Emir *et al*, 2007b; Sauer *et al*, 2009). We used antibody preparations labelled with lutetium-177 rather than radioiodine, as previous studies had suggested that anti-CA IX antibodies are deiodinated upon internalisation (Brouwers *et al*, 2004). Table 2 presents the biodistribution results for the three antibodies in LS174T tumour-bearing mice, 24 h after i.v. administration. Targeting data are expressed both as tumour/organ ratios and percent injected antibody dose per gram of tissue (% ID g^−1^)±s.e.. At this time point, SIP(L19) displayed a tumour accumulation of 9.3 %ID g^−1^, with a tumour/blood ratio of 5.8. SIP(A3) exhibited a lower tumour uptake (2.4 %ID g^−1^), but a better tumour/blood ratio (16.7). The negative control antibody displayed lower tumour uptake (1.1 %ID g^−1^), in line with previous studies (Tarli *et al*, 1999).

## Discussion

In this study, we describe the isolation of the first fully human monoclonal antibodies (A3 and CC7) with high affinity to CA IX. Furthermore, the *in vivo* distribution and tumour-targeting properties of the two antibodies have been investigated using both fluorescence-based techniques and radiolabelled antibody preparations.

Monoclonal antibodies represent the most rapidly growing sector of Pharmaceutical Biotechnology (Walsh, 2006), particularly for cancer therapy applications. As antibodies recognise their cognate antigens with exquisite selectivity, intense research efforts are devoted to the exploitation of these binding specificities for the development of superior therapeutic agents (Carter, 2006). As rodent and chimeric antibodies are immunogenic in patients, there is an increased need for good quality human monoclonal antibodies, which can be used for imaging and therapeutic purposes (Winter and Harris, 1993; Pendley *et al*, 2003; Presta, 2006). Both A3 and CC7 are fully human antibodies, which recognise human CAIX in its native conformation with affinities in the low nanomolar range. Interestingly, it was necessary to express the recombinant antigen in a mammalian system, as previous attempts to generate anti-CA IX antibodies from bacterially produced catalytic domain led to the isolation of antibody clones, which did not recognise the antigen in its native conformation (data not shown). With the newly reported A3 and CC7 antibodies CA IX was shown to be expressed heterogeneously throughout the majority of the studied tumours, with exception of renal cell carcinomas in which all cancer cells express the antigen as a consequence of a mutated *VHL* gene (Rathmell and Chen, 2008) (Figure 5). The hypoxia-related CA IX expression was usually detectable at a distance>100 *μ*m from the nearest oxygen-supplying blood vessel. This finding is in agreement with previous reports (Hockel *et al*, 1996; Wykoff *et al*, 2000, 2001; Beasley *et al*, 2001; Koukourakis *et al*, 2001; Loncaster *et al*, 2001) and in accordance with the diffusion limit of oxygen within tissues, which has been measured to be around 150 *μ*m (Folkman *et al*, 2000; Vaupel, 2004).

The A3 and CC7 antibodies do not inhibit CA IX activity and do not bind to CA XII, which is upregulated along with CA IX under hypoxic conditions and which shares only 39% sequence identity with CA IX. In general, the choice of non-inhibitory antibodies represents a useful general precaution for antibodies specific to targets (e.g., matrix metalloproteinases), where endogenous inhibitors may compromise *in vivo* localisation performance of the antibody. To implement biomedical strategies aimed at the simultaneous inhibition of CA IX and CA XII activities Chiche *et al*, 2009 (*Cancer Res.*, 69, 358–368), the use membrane-impermeable sulfonamides, which display inhibitory constants in the low nanomolar range both towards CA IX and CA XII may be preferable (Svastová *et al*, 2004; Cecchi *et al.*, 2005; Dubois *et al*, 2007; Supuran, 2008; Ahlskog *et al*, 2009).

By multi-fluorescence microscopy we subsequently demonstrated that the A3 and CC7 antibodies, in recombinant SIP format, preferentially localised to hypoxic areas of LS174T and SW1222 colorectal tumour xenografts 6 h after i.v. administration. The tumour uptake was dramatically more efficient compared with normal organs, which displayed minimal staining at the same time point, using an immunofluorescence detection method (Figure 7).

In the LS174T model an excellent overlap was observed between pimonidazole staining and structures targeted *in vivo* by the anti-CA IX antibodies in all studied animals, although A3 and CC7 appeared to stain slightly closer to the vasculature than did the pimonidazole. The targeted structures were furthermore superimposable to the staining pattern of an *ex vivo* applied commercially available polyclonal anti-CA IX antiserum. In the SW1222 model CA IX expression was consistently detected in a much broader area with the A3 and CC7 antibodies compared with the one covalently modified by pimonidazole (Figure 8 and Supplementary Figure). In these tumours, both A3 and CC7 showed reduced accumulation compared with the *ex vivo* applied commercial anti-CA IX antiserum, and were in close proximity to both large and small vascular structures. This may indicate a barrier preventing a homogenous targeting of antigen within the tumour mass, although other tumour factors may also be involved. Interestingly, these large vascular structures were only poorly perfused with the Hoechst dye.

To our knowledge, this is the first study in which pimonidazole staining, CA IX targeting (both *in vivo* and *ex vivo*) and perfusion are simultaneously analysed. As the time points for the injection of antibody, pimonidazole and Hoechst 33342 were different (6 h, 30 min and 1 min before killing, respectively), we cannot exclude that a transient occlusion of certain vascular structures took place (Chaplin *et al*, 2006).

Limitations in antibody diffusion within solid tumour masses have previously been reported for reagents used in the IgG format (Adams and Weiner, 2005; Dennis *et al*, 2007; El Emir *et al*, 2007a), mainly in relation to the so-called ‘antigen barrier’. In this study, we have used antibodies in SIP format (Borsi *et al*, 2002; Villa *et al*, 2008), as this format and similar mini-antibody formats (Wu and Olafsen, 2008) have extensively been shown to offer distinctive advantages both for imaging applications (Leyton *et al*, 2009); von Lukowicz *et al*, 2007; Wei *et al*, 2008) and for radioimmunotherapy of cancer (Berndorff *et al*, 2005; Tijink *et al*, 2006; Kenanova *et al*, 2007; Sauer *et al*, 2009). Recent publications suggest that smaller high-affinity ligands (MW < 2000 Da) may enjoy a much more rapid tissue distribution compared with antibodies and antibody fragments (Low *et al*, 2008). High-affinity low-molecular weight ligands to CA IX have been reported (Supuran, 2008) and it will be interesting to compare their *in vivo* tumour targeting properties with those of the human antibodies A3 and CC7 with a molecular weight of 76 kDa.

In our biodistribution studies we have used lutetium-177 as radionuclide, in light of previous reports indicating that dehalogenation takes place with internalising anti-CA IX antibodies (Brouwers *et al*, 2004). With this radiometal, we observed high kidney and liver values (Table 2), similar to what we had previously reported for the ^177^Lu-labelled L19 antibody in SIP format (Tijink *et al*, 2006). In the pairwise comparison presented in this article, L19 appeared to yield higher tumour uptake values, yet at the expense of a slower blood clearance. Tumour pre-targeting strategies could also be considered, as they have yielded excellent tumour/organ ratios over a range of time points after injection (van Schaijk *et al*, 2005). By contrast, Fab and F(ab')_2_ preparations of the same antibody did not exhibit improved selectivity compared with the IgG format (van Dijk *et al*, 1991).

Our group has developed and brought to clinical trials human monoclonal antibody derivatives based on the L19 (Pini *et al*, 1998), F8 (Villa *et al*, 2008) and F16 (Brack *et al*, 2006) antibodies, specific to splice isoforms of fibronectin and of tenascin-C, respectively. These antibodies display comparable *in vivo* biodistribution results and can target a broad variety of tumours. However, their uptake within the solid tumour mass is confined to the subendothelial extracellular matrix (Demartis *et al*, 2001; Borsi *et al*, 2002; El-Emir *et al*, 2007b; Villa *et al*, 2008). The human anti-CA IX antibodies appear to target a similarly broad spectrum of cancers, yet with a four-times lower tumour uptake and a broader tissue distribution within the lesion. For this reason, one could envisage the simultaneous use of vascular-targeting and hypoxia-targeting antibodies for pharmacodelivery applications, to achieve a more homogenous distribution to a therapeutic agent (e.g., cytotoxic drug; (Wu and Senter, 2005; Carter and Senter, 2008)) within the tumour mass.

## Figures and Tables

**Table 1:** Relevant amino acid positions of antibody clones isolated from the designed synthetic libraries

	**VH chain**			**VL chain**		
**ScFv**	**31–33^a^**	**52–56^a^**	**95–100^a^**	**31–32^a^**	**50–53^a^**	**91–96^a^**
A11	S Y A	S G S G G S	G K W R T D	S Y Y	G K N N	P R G G R D
A3	**W Y A**	S G S G G S	G K W R T D	**R H L**	G K N N	P R G G R D
CC7	**W Y A**	**A G T** G G **H**	G K W R T D	**R H L**	G K N N	P R G G R D

Positions that are mutated in the primary antibody library (ETH-2-Gold) are underlined. Residues in A3 and CC7, mutated during the affinity maturation procedure, are in boldface. CC7 revealed an additional mutation (R instead of K) at position 39 in the VL chain sequence. Single amino acid codes are used according to standard IUPAC nomenclature.

a Numbering according to (Tomlinson *et al*, 1992) and (Williams *et al*, 1996).

**Table 2:** Biodistribution experiments of ^177^Lu-labelled anti-CA IX antibodies in nude mice bearing LS174T human colorectal adenocarcinoma xenografts

	**SIP(A3)**	**SIP(HyHEL-10)**	**SIP(L19)**
*Organs*			
Tumour	**1.0** (2.41±0.19)	**1.0** (1.13±0.08)	**1.0** (9.30±1.11)
Blood	**16.7** (0.14±0.03)	**12.5** (0.09±0.01)	**5.8** (1.59±0.11)
Liver	**0.2** (11.83±0.50)	**0.1** (15.91±0.83)	**0.6** (16.08±0.68)
Lung	**2.4** (0.99±0.06)	**1.1** (1.03±0.09)	**4.0** (2.33±0.13)
Spleen	**0.5** (5.20±0.50)	**0.0** (28.54±7.12)	**1.3** (6.95±0.35)
Heart	**2.4** (0.99±0.07)	**0.9** (1.27±0.07)	**4.8** (1.93±0.05)
Kidney	**0.0** (85.72±5.67)	**0.1** (12.14±0.62)	**0.5** (20.67±0.24)
Intestine	**2.7** (0.88±0.02)	**2.3** ( (0.49±0.04)	**3.5** (2.68±0.27)

Tumour/organ ratios, at 24 h after i.v. injection, are indicated in boldface. The numbers in brackets correspond to the percent injected antibody dose per gram of tissue (% ID/g)±s.e.

Student's *t*-test was applied to calculate significant differences. The tumour uptake of SIP(A3) is significantly higher than the one of the anti-lysozyme antibody SIP(HyHEL-10) (*P*<0.001).
