# Supplementary figures and images for: Human monoclonal antibodies targeting carbonic anhydrase IX for the molecular imaging of hypoxic regions in solid tumours
Source: Br J Cancer. 2009 Jul 21;101(4):645–57. doi: 10.1038/sj.bjc.6605200 (PMC2736829; doi:10.1038/sj.bjc.6605200)

**Pimonidazole**

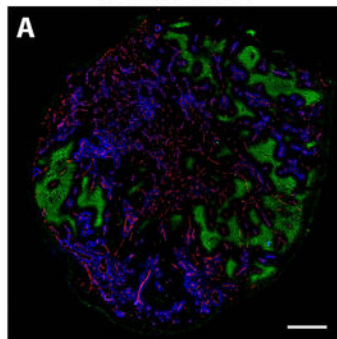

**SIP(CC7) (*in vivo*)**

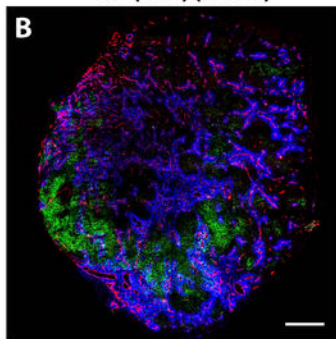

**Anti-CA IX (*ex vivo*)**

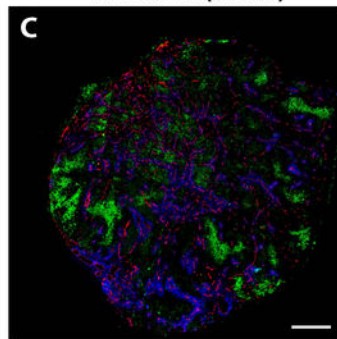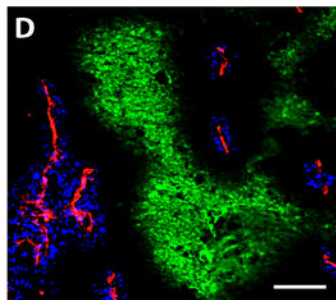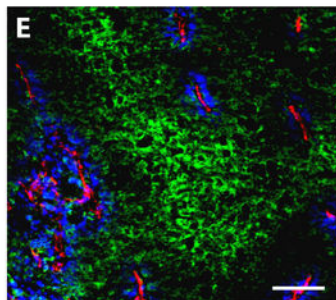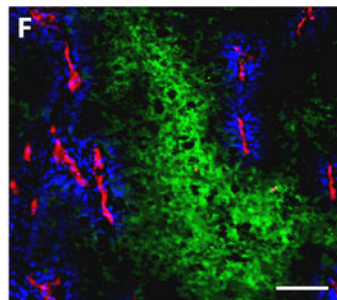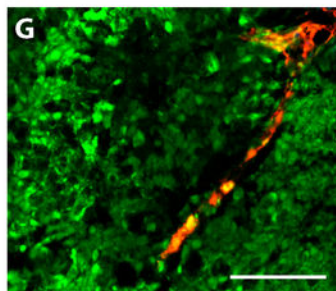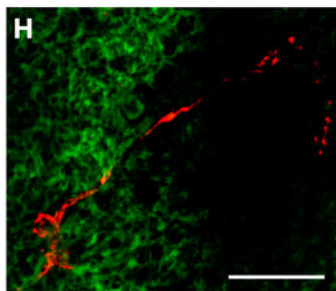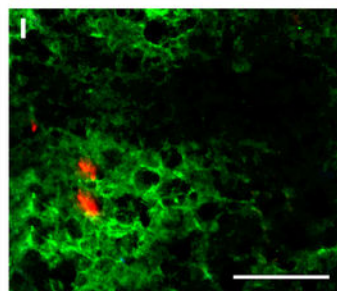

Supplement: Supplementary Figure 1 [file 6605200x1.pdf]

**Pimonidazole**

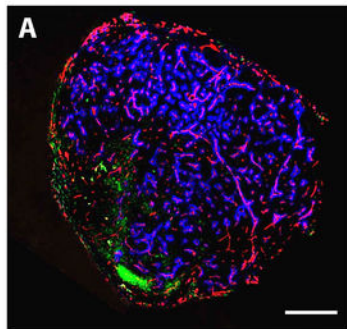

**SIP(CC7) (*in vivo*)**

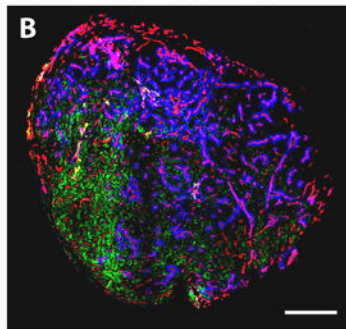

**Anti-CA IX (*ex vivo*)**

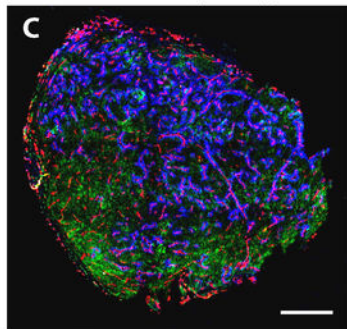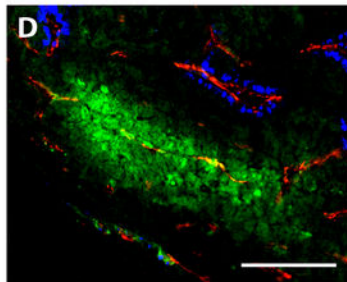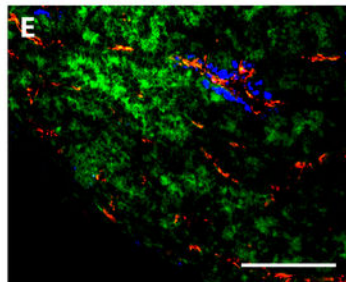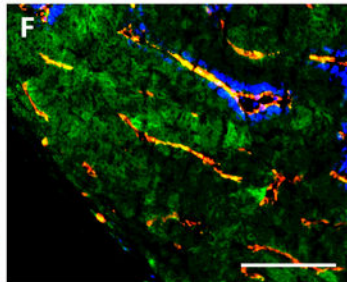

Supplement: Supplementary Figure 2 [file 6605200x2.pdf]
