# Supplementary material for: Human monoclonal antibodies targeting carbonic anhydrase IX for the molecular imaging of hypoxic regions in solid tumours
Source: Br J Cancer. 2009 Jul 21;101(4):645–57. doi: 10.1038/sj.bjc.6605200 (PMC2736829; doi:10.1038/sj.bjc.6605200)
Supplement: Supplementary Figure Legends [file 6605200x3.doc]

Supplementary Figure 1: Multi-fluorescence microscopy analysis in LS174T xenograft-bearing mice. (**A**, **B**) Representative overlays of multiple digital fluorescence images of a LS174T tumour injected with pimonidazole [30 min prior to sacrifice] and SIP(CC7) [30 min prior to sacrifice] demonstrating perfusion with Hoechst 33342 [1 min prior to sacrifice] (blue), blood vessel staining (red), pimonidazole binding (green, left panel) and CA IX targeting by SIP(CC7) (green, middle panel). (**C**) *Ex vivo* staining of an adjacent tumour section with a polyclonal anti-CA IX antiserum. (**D**-**F**) Higher magnification images of mainly perfused areas of the corresponding tumour sections. (**G**-**I**) Higher magnification images of mainly CA IX positive areas of the corresponding tumour sections. (**A**-**C**) Scale bar = 500 μm and (**D**-**I**) scale bar = 100 μm.

Supplementary Figure 2: Multi-fluorescence microscopy analysis in SW1222 xenograft-bearing mice. (**A**, **B**) Representative overlays of multiple digital fluorescence images of a SW1222 tumour injected with pimonidazole [30 min prior to sacrifice] and SIP(CC7) [6 h prior to sacrifice] demonstrating perfusion with Hoechst 33342 [1 min prior to sacrifice] (blue), blood vessel staining (red), pimonidazole binding (green, left panel) and CA IX targeting by SIP(CC7) (green, middle panel). (**C**) *Ex vivo* staining of an adjacent tumour section with a polyclonal anti-CA IX antiserum. (**D**-**F**) Higher magnification images of mainly perfused areas and CA IX positive areas of the corresponding tumour sections. (**A**-**C**) Scale bar = 500 μm and (**D**-**F**) scale bar = 100 μm.
